# Understanding Cancer-Related Pain: Pathophysiology, Classification, and Treatment Modalities

**DOI:** 10.7759/cureus.91395

**Published:** 2025-09-01

**Authors:** Jerish Murari, Ish Sharma, Siddharth Arjun Atwal, Aparna Sharma, Sukanta Bandyopadhyay, B. Shalini, Manish Kumar

**Affiliations:** 1 Department of General Medicine, Chettinad Hospital and Research Institute, Kelambakkam, IND; 2 Department of Ayurvedic Pathology, Babe Ke Ayurvedic Medical College and Hospital, Daudhar, IND; 3 Department of Emergency Medicine, Civil Hospital Malerkotla, Malerkotla, IND; 4 Department of Ayurvedic Eye and Ear, Nose, and Throat (ENT), National Institute of Ayurveda, Jaipur, IND; 5 Department of Biochemistry, Rama Medical College Hospital and Research Centre, Kanpur, IND; 6 Department of Pediatric Medicine, National Institute of Siddha, The Tamil Nadu Dr. M.G.R. Medical University, Chennai, IND; 7 Department of Microbiology, School of Science, YBN University, Ranchi, IND; 8 Department of Cancer Biology, ESIC Medical College and Hospital, Ranchi, IND

**Keywords:** artificial intelligence, cancer-related pain, mechanism-based treatment, precision oncology, translational pain research

## Abstract

Cancer-related pain (CRP) is a complex, multidimensional challenge in oncology that undermines quality of life, psychological well-being, and treatment adherence. This narrative, mechanism-informed review synthesizes pathophysiology, classification systems, and multidisciplinary management strategies to provide clinical insights and research priorities. It connects nociceptive, neuropathic, and mixed pain mechanisms to practical interventions, emphasizing peripheral and central sensitization in chronicity. Major frameworks, including the WHO analgesic ladder, International Classification of Diseases, 11th Revision, Edmonton Classification System for Cancer Pain, and European Pain Federation standards, are appraised alongside assessment tools such as the Visual Analogue Scale, Brief Pain Inventory, and Hospital Anxiety and Depression Scale, with examples of their clinical application. Management is framed within a flexible, mechanism-based, multimodal model that integrates pharmacologic, adjuvant, interventional, and psychosocial approaches, delivered through coordinated, multidisciplinary teams. Evidence for complementary modalities, such as acupuncture and mindfulness-based stress reduction, remains preliminary and heterogeneous, requiring further high-quality trials, whereas opioid-based pharmacologic approaches and structured psychosocial interventions such as cognitive behavioral therapy are supported by more robust, established evidence. Similarly, innovations like AI-driven monitoring and pharmacogenomics hold promise but are still in the early validation phase, underscoring the need to distinguish between evolving and well-established domains of cancer pain management. The principal actionable priorities are to adopt mechanism-based classification, embed multidisciplinary collaboration, expand multimodal access in low-resource settings, and rigorously validate emerging pharmacogenomic and digital health innovations before widespread clinical integration.

## Introduction and background

Cancer-related pain (CRP) is one of the leading and most difficult complications of malignancy, which is experienced by more than 60% of patients in the course of the disease and up to 80% in advanced cases [1]. This burden notwithstanding, management is not uniform, especially in low- and middle-income countries (LMICs), where approximately 70% of patients get suboptimal treatment [2]. Causative factors are strict opioid policies, the lack of trained pain experts, the cultural stigmas against reporting pain, and underfunded healthcare systems that do not allow pain to be consistently measured and treated. The review focuses on such disparities in a global context, identifying scalable, locally relevant solutions, including access to essential medicines, patient education, and culturally adapted and multidisciplinary training based on available local resources. The problem of under-treatment also takes place in high-income contexts, which is caused by the lack of consistency in pain assessment, diversity in the level of expertise of clinicians, and unwillingness to prescribe opioids due to existing recommendations of the WHO, European Pain Federation, and American Society of Clinical Oncology [3]. The etiology of CRP is multifactorial and includes tumor invasion, injury caused by treatment (e.g., surgery, radiotherapy, and chemotherapy), and comorbidities [4]. CRP, when poorly managed, will lead to physical debility, emotional distress, decreased adherence to treatment, and poor quality of life [5].

CRP is not only a physical phenomenon but also a complex of interactions between biological, psychological, and sociocultural factors [6]. These determinants are also considered in this review as the most prominent modulators of pain perception and response to treatment and are directly integrated into the suggested management framework in the form of culturally adapted communication, psycho-oncology assistance, caregiver involvement, and policy-based interventions that target structural impediments to equal pain management. That is why this multidimensionality causes the fact that the uniform treatment algorithms can be efficient in some patients but not enough in others. Two patients of similar disease progression and tumor load may differ significantly in the course of pain development due to previous trauma, coping abilities, support system, and cultural perception of pain expression [7]. Pain may continue to occur following tissue healing as a result of central sensitization and maladaptive neuroplasticity and may be complicated by comorbid anxiety or depression [8]. To effectively address these drivers, patient-specific, adaptive interventions that combine neurobiological treatment with psychosocial support, patient education, and culturally competent care, approaches that have not been fully embraced by mainstream oncology, are needed.

Although the field of oncology has improved, pain management has not improved due to knowledge deficits in neurobiology, varied implementation of classification schemes, limited opioid availability, and limited acceptance of new modalities [9]. Evidence-based guidelines are clear and provided by the National Comprehensive Cancer Network (NCCN) Guidelines for Adult Cancer Pain (2025), the WHO cancer pain guidelines (2023 update), and the European Society for Medical Oncology (ESMO), although they are not completely adhered to. Multinational audits reveal that less than 60% of eligible patients are receiving guideline-concordant opioid prescribing and that pain assessment and documentation vary significantly [6]. This persistent implementation gap emphasizes the significance of strategies that may fill in the gaps between guidelines and practice. CRP has been discussed in literature in silos, mechanisms, pharmacologic care, or psychosocial support instead of being integrated into comprehensive, patient-centered models. Multidisciplinary integrative oncology programs in Canada and the Netherlands have demonstrated 20-30% reductions in pain scores and enhanced functioning [10], and Australian nurse-led digital pain monitoring has also led to greater adherence to analgesic regimens and fewer unplanned hospitalizations [11]. New modalities, such as integrative oncology, digital monitoring, and precision medicine, have promise but must be validated, cost-effectively evaluated, and applied in a manner that is equitable.

The realization that CRP is a multidimensional phenomenon requires clinicians to step out of the strict protocols and use a more personal, biopsychosocial approach. This implies the incorporation of pharmacologic, psychological, and culturally sensitive interventions in care plans, particularly in a place where there is limited access to specialized pain services. Psychosocial factors and biological drivers should be assessed by clinicians on a regular basis to inform individual treatment pathways.

**Figure 1:**
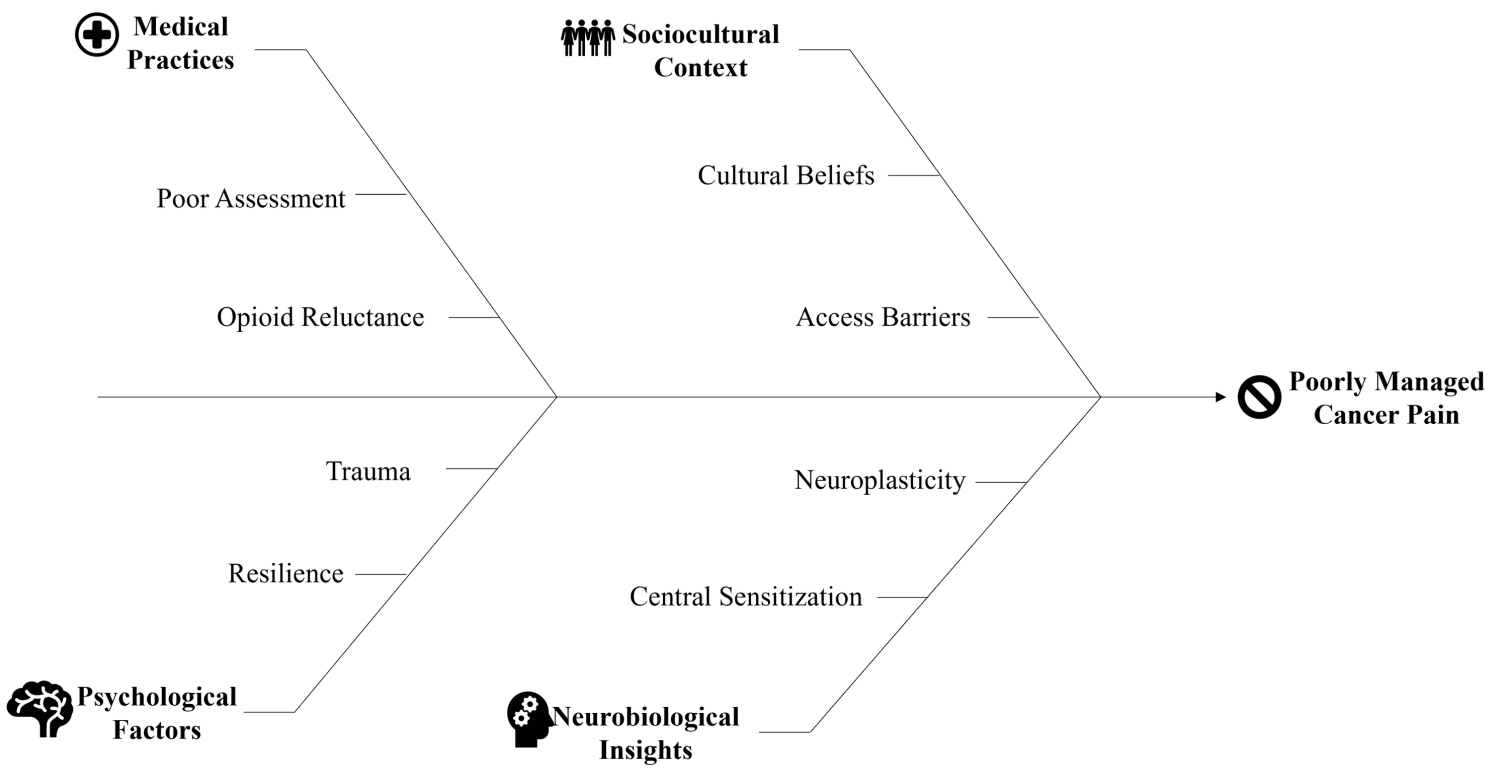
Multifactorial determinants of poorly managed cancer pain Image credit: Jerish Murari

In contrast to previous reviews that selectively focus on isolated components of CRP, the article offers a critically appraised, mechanism-linked synthesis that links biological pathways, classification systems, pharmacologic and nonpharmacologic alternatives, and emerging technologies such as pharmacogenomics and AI-based applications. Mechanistic classification, multidisciplinary models of management, evidence-based pharmacologic and psychosocial interventions, and context-specific adaptations to low-resource settings are given particular depth, with shorter descriptions of other emerging fields such as digital health and new molecular therapies. Both high- and low-resource settings, pediatric and geriatric populations, and management strategies are taken into consideration, and mechanistic insights are directly connected with real-life clinical decision-making. The review is based on the latest frameworks of guidelines and practice-related data and also evaluates emerging innovations with equal concern for opportunities and shortcomings. Initial-stage studies of AI-based pain management have demonstrated better-timed analgesic modification and patient-reported control, and pharmacogenomic characterization of opioid metabolism genes (e.g., OPRM1 and CYP2D6) holds potential to optimize dosing and minimize adverse effects. Results are still preliminary, and small samples and brief follow-up and limited data on cost-effectiveness; large, multicenter studies are required to validate and apply. In combination, the review provides practical recommendations to clinicians, research priorities, and policy recommendations to promote equitable and mechanism-driven management of CRP (Figure 1).

Objectives of the review

The purpose of this review is to provide an integrated, mechanism-informed synthesis of existing evidence on CRP, encompassing pathophysiology, classification systems, and pharmacologic and nonpharmacologic management. Beyond summarizing established knowledge, it aims to critically appraise methodological quality, identify evidence gaps, and highlight the clinical relevance of emerging fields such as integrative oncology and digital health technologies. Particular emphasis is placed on linking mechanistic insights to practical decision-making and adapting strategies to diverse care settings, from high-resource institutions to LMIC contexts. In this wide range, the review focuses on clinically feasible approaches, including mechanism-based categorization to move toward individualized care, scalable multidisciplinary models to address resource-constrained contexts, and the aggressive testing of new opportunities like pharmacogenomics and AI-based monitoring to guarantee that innovative knowledge translates into concrete improvements in patient outcomes. The overarching goal is to enhance diagnostic precision, optimize individualized care, and improve quality of life for people living with CRP by connecting current clinical evidence to future research priorities and implementation strategies.

Methodology

The narrative review method was selected to identify established practices and emerging practices not yet reflected in large randomized trials. The literature was retrieved from PubMed, Google Scholar, and ScienceDirect (2015-2025), and classic previous literature. The results were matched against the most relevant clinical practice guidelines, the NCCN Guidelines for Adult Cancer Pain (2025), the WHO Cancer Pain Guidelines (2023), and the ESMO recommendations to correlate the findings with authoritative guidelines. The search strategy was designed to utilize controlled vocabulary (e.g., MeSH terms) and free-text keywords (e.g., cancer-related pain, mechanism-based management, pharmacogenomics, integrative oncology, digital pain monitoring, and pediatric cancer pain) and was refined using Boolean operators and supplemented by screening reference lists. Relevant titles and abstracts were identified, and full texts were reviewed by consensus resolution. The quality and concordance of the guideline and real-world applicability of the evidence were appraised.

Digital pain tracking, pharmacogenomics, and integrative treatments were covered. Early studies of AI-based monitoring indicate faster analgesic titration, and initial pharmacogenomic models indicate the possible utility of individual opioid prescriptions, but these need to be expanded. Narrative bias risks were addressed by triangulating the sources using multidisciplinary approaches and identifying evidence gaps. Implementation of frameworks like the International Classification of Diseases, 11th Revision (ICD-11) and Edmonton Classification System for Cancer Pain (ECS-CP) is hindered by training requirements, shortage of specialists, and lack of EHR integration; audits show an uptake of less than 50% unless education and workflow adjustment are specifically targeted.

Framework Selection and Analysis

The frameworks considered in the present review, the WHO analgesic ladder, ICD-11, ECS-CP, and the biopsychosocial model, were chosen because of their popularity in oncology guidelines, abundance in recent publications, and relevance to clinical practice. Targeted search (2015-2025) in PubMed, Google Scholar, and ScienceDirect using both controlled vocabulary (e.g., MeSH) and free-text terms (e.g., WHO analgesic ladder, ICD-11 cancer pain, ECS-CP, and biopsychosocial model in oncology) supplemented by screening the reference lists was used to inform selection using Boolean operators. Conference proceedings and policy documents (gray literature) were also reviewed; unpublished data were not included unless peer-reviewed.

The WHO analgesic ladder has been used as a pillar in pharmacologic management, but it has been criticized for having a narrow focus on mechanism-based selection and integrative modalities. ICD-11 is less specific in diagnosis but can be applied only following the substantial training of clinicians and institutional support. ECS-CP includes psychosocial complexity assessment but is restricted mainly to specialist settings. The strength of evidence was evaluated based on the study design, sample size, methodological rigor, and conformity to guidelines, and emerging areas like pharmacogenomics and AI tools were given lower weights. We present a combined approach whereby ICD-11 aids in diagnostic grouping, ECS-CP aids in psychosocial profiles, and the WHO ladder facilitates pharmacologic escalation as part of a biopsychosocial care plan.

The Visual Analogue Scale (VAS), Brief Pain Inventory (BPI), and Hospital Anxiety and Depression Scale (HADS) are addressed in terms of measurement characteristics and their contribution to individualized care. Their validity has been established in different populations, and cross-cultural adaptation is necessary to consider linguistic, literacy, and sociocultural differences. The experience in LMICs indicates that adapted versions can be psychometrically strong, but feasibility can be limited by resource constraints, including staff training or the availability of forms. These considerations inform recommendations for context-appropriate selection and implementation. The authors developed all figures related to the framework to enhance clarity, and the interpretations were supported by existing evidence and critically appraised to be applicable in clinical situations in any healthcare setting.

## Review

Pathophysiology of CRP

CRP arises from the interplay of malignant disease and anticancer treatments, with nociceptive, neuropathic, and mixed mechanisms often coexisting to shape symptom profile and therapeutic response [9]. Mixed pain presentations occur in approximately 30-50% of CRP cases, making them a frequent and clinically significant challenge [10]. Mechanism recognition is critical for differential diagnosis: neuropathic features, such as burning or electric-shock sensations, may prompt adjuvants like gabapentinoids or serotonin-norepinephrine reuptake inhibitors (SNRIs), whereas nociceptive pain from inflammation responds better to NSAIDs and opioids. For mixed pain, the NCCN (2025) and WHO (2023) recommend combination regimens with mechanism-specific agents and nonpharmacologic modalities [11]. This classification also guides assessment using tools such as the DN4 questionnaire for neuropathic features and the VAS or BPI for severity and function [12]. Clinically, bone metastases compressing nerve roots require dual targeting of somatic nociception and neuropathic transmission to avoid undertreatment despite guideline adherence.

Nociceptive pain, whether somatic (e.g., periosteal stretch in bone metastases) or visceral (e.g., pancreatic tumor infiltration), arises from tissue injury and inflammatory mediators [11]. Neuropathic pain may result from tumor infiltration or treatment-related injury such as chemotherapy-induced peripheral neuropathy [13]. Chronic CRP persists via TRPV1-mediated sensitization, dorsal horn hyperexcitability, reduced descending inhibition, and glial activation [14]. Addressing central sensitization is therefore essential. Evidence-based strategies include SNRIs, tricyclic antidepressants, CBT, mindfulness-based stress reduction, and neuromodulatory techniques like TMS or spinal cord stimulation [13-15], as endorsed by NCCN (2025) and ESMO (2024). Screening with the CSI and integrating psycho-oncology and rehabilitation into multidisciplinary teams, including scalable options such as telehealth CBT, can promote equitable, mechanism-aligned care.

Integrated classification approach

The most pragmatic system is a mechanistic-temporal one, which separates acute (usually procedural or treatment-related) and chronic (more than three months) pain and subtypes mechanisms as nociceptive, neuropathic, or mixed [15]. This dual lens enhances the accuracy of treatment delivery over both strictly temporal and symptom-based systems. The mechanistic-temporal model contrasts with the WHO analgesic ladder, which focuses on the pharmacologic escalation of pain, or ICD-11, which provides diagnostic specificity with no direct guidance on management, in that it enables the real-time selection of both pharmacologic and nonpharmacologic approaches, based on the subtype and duration of pain [7]. This is of specific concern to breakthrough cancer pain (BTcP), transient flares that can affect up to 70% of patients, and is the most underdiagnosed since it is episodic and inconsistently defined. Rapid-onset opioids such as transmucosal fentanyl may be useful in some circumstances, and there is proven efficacy in high-resource environments, but little accessibility and affordability in most jurisdictions [16]. Areas of research priority involve comparative studies of optimal agent choice, nonopioid adjuncts, and adaptation of BTcP protocols to low-resource settings. This mechanism-based classification aligns with NCCN (2025), WHO (2023), and NCI PDQ (2025) recommendations, which are increasingly seeing mechanistic profiling as the key to precision pain management [9].

CRP can be either tumor-related (e.g., bone metastases or nerve invasion) or treatment-induced (e.g., surgical neuropathy or radiation fibrosis) [17]. Other systems, such as ICD-11 or ECS-CP, already incorporate temporal, mechanistic, and psychosocial components; however, training and implementation challenges are problematic [18]. Canadian and Dutch pilot programs raised uptake by incorporating framework training in oncology residencies, integrating fields in electronic health records, and using nurse-led screening, and an Australian palliative care network increased the use of ECS-CP >40-fold via multidisciplinary workshops and decision-support prompts [11]. The strategies show how the use of such frameworks in everyday workflows can benefit the mechanism-based profiling and clinical decision-making. Nevertheless, the absence of trained personnel and incorporation into the electronic health systems have been key obstacles in LMICs, which further solidifies disparities in the adoption of mechanistic frameworks around the world. Early screening and assessment of mixed-mechanism pain, peripheral and central-focused therapeutic interventions, and multidisciplinary management of physical, psychological, and social aspects should be recommended to clinicians. Table 1 shows that cancer pain assessment is multidimensional, integrating clinical, psychological, and mechanistic tools to guide individualized management.

**Table 1:** Multidimensional assessment tools and modalities for CRP BPI, Brief Pain Inventory; CBT, cognitive behavioral therapy; CRP, cancer-related pain; CSI, Central Sensitization Inventory; HADS, Hospital Anxiety and Depression Scale; NCCN, National Comprehensive Cancer Network; NRS, Numeric Rating Scale; PCS, Pain Catastrophizing Scale; VAS, Visual Analogue Scale

Assessment domain	Tools/methods	Purpose	References
Unidimensional scales	NRS, VAS	Quick quantification of pain intensity; most useful for nociceptive pain severity	[2]
Continuity of assessment	Repeated evaluations over the disease trajectory	Supports adaptive treatment strategies based on clinical progression	[2]
Neurophysiologic testing	Electrophysiological studies	Clarifies nerve injury type (peripheral vs. central origin); recommended for neuropathic subtyping	[4]
Psychosocial integration	CBT, psycho-oncology support	Addresses emotional comorbidities influencing pain management	[4]
Equity considerations	Simplified pain scales (VAS, Faces Pain Scale), caregiver-assisted reporting	Necessary alternatives in LMICs where advanced imaging or neurophysiologic testing are not accessible	[7]
Mechanism-specific screening	DN4 Questionnaire, PainDETECT, CSI	Recommended by NCCN (2025) for routine neuropathic and central sensitization assessment in cancer pain	[9]
Imaging	MRI, CT scans	MRI for spinal/nerve involvement; CT for bone/visceral pathology; aligns with nociceptive etiology confirmation	[10]
Clinical history and interview	Onset, location, intensity, quality, pattern, past interventions	Foundational step for individualized pain characterization; key to distinguishing nociceptive vs. neuropathic descriptors	[11]
Multidimensional tools	BPI	Assesses pain severity and interference with sleep, mood, mobility, etc.; helpful for mixed pain profiles	[13]
Patient-reported outcomes	McGill Pain Questionnaire, EORTC QLQ-C15-PAL	Captures sensory, emotional, and temporal pain attributes; McGill can distinguish neuropathic from nociceptive descriptors	[13]
Team-based approach	Palliative specialists, psychologists, physiotherapists, and social workers	Enables dynamic, multidisciplinary care aligned with evolving patient needs	[14]
Psychological assessment	HADS, PCS	Detects depression, anxiety, and catastrophizing that may amplify pain perception	[15]
Physical examination	Sensory testing, allodynia, and motor function	Identifies neuropathic features like sensory loss or weakness	[17]

Table 2 shows that CRP can be systematically classified into nociceptive, neuropathic, and mixed mechanisms, with each category linked to characteristic diagnostic clues, validated assessment tools, and targeted treatment strategies. This framework supports mechanism-based, individualized management in line with current international guidelines.

**Table 2:** Mechanism-based framework for CRP assessment and management BPI, Brief Pain Inventory; CRP, cancer-related pain; DN4, Douleur Neuropathique en 4 Questions; SNRI, serotonin-norepinephrine reuptake inhibitor; TCA, tricyclic antidepressants; VAS, Visual Analogue Scale

Pain mechanism	Diagnostic clues	Assessment tools	Targeted treatments	Notes	References
Nociceptive	Dull, aching, localized; related to tissue injury, inflammation, or tumor mass effect	Clinical history and exam, VAS, BPI	NSAIDs, opioids, corticosteroids, bone-targeted agents	Typically well-controlled with standard analgesics when recognized early	[2,11]
Mixed	Combination of nociceptive and neuropathic features; fluctuating or breakthrough pain patterns	Comprehensive multidimensional assessment (BPI and McGill), repeated evaluations	Combination regimens (opioids + adjuvants), flexible opioid rotation, psychosocial therapies	Most common presentation (30-50% of CRP); requires mechanism-guided multimodal care	[10]
Neuropathic	Burning, shooting, electric-shock sensations; allodynia, sensory loss, motor weakness	DN4 questionnaire, PainDETECT, sensory exam, neurophysiologic testing	TCAs, SNRIs, gabapentinoids, opioids, nerve blocks	Often requires adjuvants and/or interventional approaches; guideline-recommended screening is underutilized	[13]

Pharmacologic management of CRP

Although the WHO analgesic ladder continues to be a pillar in the treatment of CRP, its historical symptom-severity orientation undervalues potential mechanism-based targeting and incorporation into nondrug approaches [19]. In contemporary practice, it is supposed to be a dynamic, two-way instrument, which can be escalated or de-escalated depending on pain mechanism, comorbidities, and patient goals. This is confirmed by real-world data: a mechanism-based model in which neuropathic characteristics led to earlier adjuvant or interventional treatment decreased uncontrolled pain by 18% and opioid-related adverse events by 12% compared with inflexible ladder adherence in the Netherlands [20]. In a tertiary cancer center in India, allowing for early use of strong opioids in severe pain due to bone metastases resulted in a 25% improvement in pain scores without more misuse [21]. In LMICs, ladder adherence may be costly and lead to delays in strong opioids, which demonstrates the necessity of local adaptations. Structured assessment must initiate mechanism-based tailoring, including the use of tools like the DN4 questionnaire for neuropathic characteristics and the BPI to assess functional impact to promote correct subtype identification and specific intervention. In practice, opioids remain the mainstay for nociceptive pain, tricyclic antidepressants/SNRIs/gabapentinoids are preferred for neuropathic features, and combination regimens are essential for mixed mechanisms, as endorsed by current guidelines [22].

First-line opioids (morphine, oxycodone, and transdermal fentanyl) are still needed, but interpatient pharmacogenomic variability frequently requires opioid rotation [23]. In the case of BTcP, the use of rapid-onset fentanyl is effective but restricted by cost, availability, and inconsistent definitions [24]. Tricyclic antidepressants and SNRIs are adjuvants with a positive effect on neuropathic pain [25], whereas the relatively novel group of gabapentinoids, despite mechanistic plausibility, shows no strong cancer-specific evidence, with small, short-term trials having excluded advanced disease [23]. In certain syndromes, corticosteroids provide anti-inflammatory effects [26], and bone-targeted agents decrease skeletal events with a modest analgesic effect [27]. Thus, while opioids and antidepressants are supported by robust evidence, gabapentinoids and bone-targeted agents remain supported only by modest or preliminary data.

**Figure 2:**
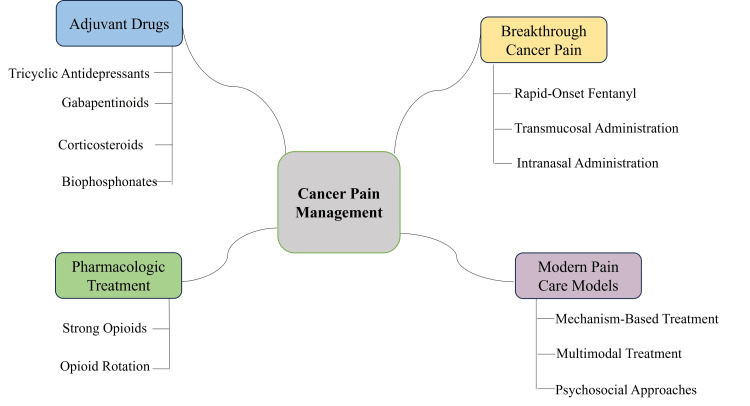
Core pillars of cancer pain management: pharmacologic, adjuvant, and integrative strategies Image credit: Jerish Murari

Multimodal, mechanism-driven treatment tailored to pathophysiology, patient preferences, and resource context is recommended by NCCN (2025), ESMO (2024), and WHO (2023) guidelines [26]. In refractory neuropathic pain with concurrent nociceptive bone metastases, these guidelines advocate optimizing opioids for nociceptive control, adding duloxetine or gabapentinoids for neuropathic elements, and considering bisphosphonates, denosumab, or interventional procedures. In LMICs, multicenter studies show that cost-effective regimens such as immediate-release oral morphine plus amitriptyline for neuropathic components can match the efficacy of higher-cost alternatives at a fraction of the cost [28]. Yet equitable access remains hindered by restrictive opioid regulations, complex licensing, supply chain inconsistencies, insufficient clinician training, and cultural norms discouraging pain reporting [29]. India’s 2014 amendment to the Narcotic Drugs and Psychotropic Substances Act streamlined licensing and increased morphine availability in palliative programs without evidence of misuse [30]. The WHO’s data show that in most African and Southeast Asian nations, per capita opioid use is less than 1% that of high-income nations despite similar need [9], underlining the need to reform regulation, clinician training, and supply-chain optimization, as well as cost-effective pharmacologic solutions. Such a dramatic difference highlights the importance of adjusting guideline recommendations to context through prioritizing low-cost essential medications in LMICs and leaving high-cost adjuvants and fentanyl formulations out of reach [31]. Figure 2 illustrates that cancer pain management integrates pharmacologic, adjuvant, breakthrough pain, and modern multimodal approaches.

Interventional approaches

Interventional procedures offer local palliation where systemic therapy is ineffective or intolerable. In well-localized pain with a clear neuroanatomic target and intact patient functionality, they are advised in refractory pain with a good prognosis (e.g., targeted nerve blocks such as celiac and superior hypogastric) [32]. They work best on neuropathic pain caused by nerve invasion or compression, in which case systemic pharmacologic approaches are not adequate. In diffuse pain that is unresponsive to systemic regimens, neuraxial delivery of drugs may be considered in situations where the side effects of the systemic regimen are limiting [33]. Neurolysis and ablation are best in patients with localized, procedure-responsive syndromes; shorter prognoses; or where relief is of paramount importance to nerve preservation in the long term [27]. Celiac plexus block has a 70-90% success rate in cancer pain of the upper abdomen [4]; block of the superior hypogastrium is useful in pain of the pelvis [34]. There should be a balance of risks and benefits, particularly in diffuse disease.

Neurolytic procedures are generally reserved for poor-prognosis cases [35]. Intrathecal drug delivery has the potential to decrease systemic toxicity [30]; however, the cost of devices and follow-up restrictions limit their application in low-resource settings [31]. The nociceptive pain of the bone is the main focus of radiofrequency ablation (RFA) and vertebroplasty, which are long-lasting and offer mobility enhancement in spinal lesions [32]. It is crucial to be involved in care planning early and be coordinated by a multidisciplinary team to achieve the best outcomes [36]. The interventional modalities can be viewed as a more upstream intervention than a last-line rescue, especially in mixed or refractory pain.

Nevertheless, early referral is frequently impeded by a shortage of interventional experience in community oncology, ignorance of the existing procedures, and a lack of access to specialists due to logistical impediments. Such issues are compounded in LMICs, where interventional pain specialists and imaging-guided therapy are limited. Means to combat these are the incorporation of interventional pain consultation pathways into the protocols of oncology care, telementoring of community clinicians, and referral networks to regional centers with the capacity to provide procedures.

Radiation and surgical pain management

Palliative radiotherapy effectively reduces pain from bone metastases and compressive lesions, often achieving meaningful relief within one to two weeks of initiation. In patients with limited life expectancy, especially those with focal, refractory pain despite optimized pharmacologic therapy, radiotherapy can provide more durable and targeted analgesia compared with escalating systemic agents, which may carry greater toxicity burdens without proportionate benefit. Short-course regimens (e.g., 8 Gy single fraction) have demonstrated comparable pain control to multi-fraction schedules, with the advantage of reduced treatment burden in frail patients [34]. Single-fraction regimens provide equivalent initial relief to multifraction schedules but have higher retreatment rates [35]. Stereotactic body radiation therapy delivers high precision in oligometastatic disease [36]. Surgical interventions, including decompressive surgery and vertebral stabilization, restore function and improve pain, particularly in spinal cord compression [37]. Patient selection based on prognosis, functional status, and goals of care is critical. Validated scoring systems such as the Tokuhashi score for spinal metastases, the Spinal Instability Neoplastic Score, or the Karnofsky Performance Status can assist in estimating survival, assessing stability, and determining suitability for surgical or radiotherapeutic interventions [38]. Table 3 illustrates that effective cancer pain management may involve tailoring radiotherapy and surgical procedures according to these prognostic insights, clinical indications, and multidisciplinary care principles.

**Table 3:** Palliative oncologic interventions for CRP management CRP, cancer-related pain; RT, radiation therapy; SBRT, stereotactic body radiation therapy

Intervention	Type	Indications	Clinical notes and evidence	References
Radiotherapy	Palliative radiation	Painful bone metastases, compressive lesions	Reduces tumor bulk, inflammation, and neural pressure	[12,13]
Implementation considerations	Multidisciplinary, goal-concordant care	Prognosis, functional status, and patient preferences	Optimal benefit when tailored to individual needs and integrated into team-based care	[12]
Colostomy/bypass surgery	Palliative visceral surgery	Obstruction due to a tumor	Restores function, indirectly reduces pain	[13]
Single-fraction RT	8 Gy in one session	Uncomplicated bone pain	Comparable pain relief to multifraction RT; higher retreatment rates	[14]
Multi-fraction RT	20-30 Gy in 5-10 fractions	Patients with a longer prognosis or spinal involvement	Selected based on performance status and logistical feasibility	[15]
Debulking surgery	Partial tumor resection	Pain from the mass effect on sensitive structures	Not curative, but often provides significant symptom relief	[15]
Spinal cord compression	Emergency radiotherapy	Neurological deficits, early intervention	Stabilization and symptom relief if treated promptly	[16]
SBRT	High-precision SBRT	Oligometastatic spinal lesions	Delivers ablative doses with minimal damage to surrounding tissue	[17]
Surgical interventions	Decompressive surgery	Spinal cord compression, intractable axial pain	Surgery + RT superior to RT alone for pain and ambulation	[19]
Vertebroplasty/kyphoplasty	Minimally invasive spine stabilization	Metastatic vertebral fractures	Rapid pain relief and mobility improvement; suitable for poor surgical candidates	[20]

Psychosocial and behavioral approaches

Psychological distress affects up to 50% of advanced cancer patients and amplifies pain perception [39]. Cognitive behavioral therapy (CBT) reduces pain intensity (standardized mean difference (SMD) ≈ -0.34), disability, and mood disturbance, partly by counteracting catastrophic thinking and central sensitization, with benefits lasting three to six months [9]. MBSR improves mood, fatigue, and pain interference. The reputed guidelines recommend CBT, MBSR, and psycho-oncology support as standard components of multimodal care. Screening with HADS or the Distress Thermometer is advised [40]. In low-resource settings, caregiver training and psychoeducation provide scalable, culturally adaptable alternatives [13].

Complementary and integrative therapies

Acupuncture, massage, yoga, Tai Chi, and music therapy are modalities of complementary and integrative medicine (CIM) that demonstrate emerging benefit [41]. The strongest randomized controlled trial (RCT) evidence is in support of acupuncture and massage, with moderate effect sizes (SMDs 2145 to 2160) lasting 12 weeks, followed by yoga and music therapy, which are supported by smaller trials, and Tai Chi, the evidence of which is yet to be established. Mechanistically, acupuncture can stimulate endogenous opioid and serotonin systems [5], massage enhances mobility and coping [42], and music therapy can modulate reward circuits that have the potential to decrease opioid use [43]. CIM is not a first-line treatment, according to the esteemed guidelines, with evidence being heterogeneous. These barriers are the training of practitioners, the diversity of methodologies, and limited access in LMICs, where CIM integration demands culturally sensitive, cost-effective models [44].

Pediatric and geriatric considerations

The assessment of pain associated with pediatric cancer (CRP) must be developmentally friendly and utilize validated tools such as the Face, Legs, Activity, Cry, Consolability (FLACC) scale and the Wong-Baker FACES Pain Rating Scale, and parents should be actively engaged in the process [45]. A number of RCTs and observational studies reveal that frequent use of such tools increases the accuracy of analgesia, decreases delays in interventions, and leads to better caregiver-clinician communication [46]. Mechanistically, pediatric pain is distinct from adults, because of immature nociceptive pathways and enhanced plasticity, which predisposes the child to central sensitization should pain not be treated adequately [47]. Tool validity is affected by cultural and linguistic considerations; cross-cultural adaptations in LMICs have been shown to be reliable provided training and validation are robust, but they are frequently constrained by literacy, human resources, and local norms [48]. Culturally tailored implementation and caregiver education are therefore critical. Opioids are to be weight-based and titrated [49], and nonpharmacologic methods like play therapy and the presence of the caregiver are also vital in settings where there are no pediatric palliative care specialists [50]. Multimodal analgesia, family-centered care, and incorporation of psychosocial support are considered standard practice according to NCCN pediatric oncology recommendations [51].

Self-report may be restricted in geriatrics by sensory or cognitive dysfunction, and measurement aids such as the Pain Assessment in Advanced Dementia (PAINAD) scale and functional behavior mapping can be used [52]. Functional mapping distinguishes between procedural, movement-related, and resting pain, which enhances opioid titration, decreases the use of inappropriate sedatives, and facilitates mobility-sparing regimens in cognitively impaired patients [53]. Mechanistically, aging is linked with nociceptive thresholds, neurodegeneration, and polypharmacy interactions that make analgesic response hard. Dosing must therefore be cautious [54], with frequent reassessment. Some ethical considerations are assent in pediatrics and advance directives in geriatrics [55]. Such complexities demand a multidisciplinary strategy and the development of adaptations to developmental or degenerative pain phenotypes based on guidelines [56]. Table 4 shows that pediatric and geriatric cancer pain management requires age-specific assessment tools, cautious pharmacologic use, supportive nondrug strategies, and ethically guided multidisciplinary care.

**Table 4:** Age-specific considerations in pediatric and geriatric cancer pain management FLACC, Face, Legs, Activity, Cry, Consolability

Domain	Pediatric population	Geriatric population	References
Ethical considerations	Require assent + parental consent; emphasis on child-centered communication	Use of surrogate decision-makers, advanced directives, and frailty-guided goal-of-care planning	[21]
Pharmacologic caution	Opioids dosed by weight; risks include respiratory depression, sedation, and gastric delay	Altered drug metabolism; risk of sedation, renal dysfunction, and polypharmacy complications	[24]
Validated tools	FLACC Scale, Wong-Baker FACES for age-appropriate assessment	PAINAD, functional behavior mapping for dementia or communication-limited patients	[26]
Assessment challenges	Nonverbal or cognitively impaired children may express pain as irritability or behavior changes	Cognitive impairment and sensory loss hinder self-reporting	[32]
Nonpharmacologic adjuncts	Play therapy, distraction, parental presence; culturally acceptable and developmentally tailored	Cognitive therapies, gentle physical activity, social engagement, and supportive care models	[57]
Team-based approach	Involves pediatric pain specialists and child psychologists	Includes geriatricians, palliative care experts, and pharmacologists for personalized care	[58]

Emerging therapies

New molecular targets include cannabinoids, sodium channel blockers, nerve growth factor (NGF) monoclonal antibodies, and TRPV1/acid-sensing ion channel (ASIC) modulators as the mechanism-based reliefs [59]. These remain in the preliminary or early validation stage. The meta-analyses of cannabinoids have yielded inconclusive results because of formulation, dosing, and study quality variability and remain subject to regulatory and standardization issues [60]. NGF antibodies (e.g., tanezumab) demonstrate promise in bone metastasis-related nociceptive pain, and selective sodium channel blockers (e.g., Nav1.7 inhibitors) target neuropathic pain processes; they are in phase II/III development in cancer and noncancer pain [61]. By contrast, TRPV1 and ASIC modulators remain largely preclinical. OPRM1, CYP450, and transporter polymorphism-based pharmacogenomic tailoring have potential as a future path to a more effective and tolerable opioid, but the data are currently restricted to small observational studies and must be validated in large multi-ethnic cohorts [62].

The technology of digital health innovations, such as the AI-based prediction of BTcP, real-time monitoring with wearable biosensors, and neuromodulation using VR, is in pilot or early-stage clinical trials [63]. Their use is limited by nonhomogeneity of evidence, lack of validation, privacy, and disparities of access, especially in LMICs. This equity gap highlights the necessity of scalable, low-cost deployment in case digital innovations will add value to global cancer pain care [64]. Recent mechanistic understanding of epigenetic regulation, endoplasmic reticulum stress, and signaling pathways, including TGF-beta and Notch, adds to these strategies and identifies potential druggable targets. The next generation of models should combine molecular precision and digital technologies with patient-centered, biopsychosocial care to guarantee that innovations can transfer into equitable and evidence-based practice and not be limited to academic or high-resource environments.

Limitations of the review

The methodology involved in this review was a narrative approach, which allows the broad involvement of various evidence at the cost of selection bias and lower reproducibility than systematic review methodologies. Although all possible attempts were made to incorporate quality and recent studies, some literature could not be included, especially in non-English languages. The included literature is mostly of high-resource origin, which can be a barrier to generalizing to low-resource contexts.

Direct comparison is difficult due to the heterogeneous designs, population, and outcome measures relied upon. There is limited or small-scale trial-based evidence on some of the emerging interventions, including cannabinoids, pharmacogenomics, and digital health tools, limiting the strength of practice recommendations. Moreover, the review summarizes previously published information and does not present new empirical data; therefore, conclusions are based on the quality and extent of the available research.

Such limitations could be countered in future systematic reviews by broadening database searches to non-English language publications, inclusion of gray literature (conference proceedings and policy reports), and use of structured search strategies with two independent screenings and standardized risk-of-bias tools. Specific interventions that focus on collecting LMIC data, including the use of regional databases, partnerships with local researchers, and the use of context-specific outcome measures, would increase global representativeness and equity in the evidence base.

Future recommendations

The future of CRP management should be based on a concerted effort between research, clinical care, and policy, with a particular emphasis on mechanistic classification systems that incorporate temporal, etiological, and functional levels to inform specific treatment. Examples of pilot programs have included ECS-CP integration in Canada, ICD-11 implementation in the Netherlands, and a multicenter effort in Australia that integrates both mechanistic subtyping with temporal profiling, which has demonstrated feasibility with the support of training, workflow integration, and enabling policy. Pharmacologic, interventional, and integrative approaches should be compared across a wide range of populations, such as pediatric and low-resource populations, through large multicenter trials. The personalization of analgesia is possible with pharmacogenomic profiling of opioid receptors, metabolic enzymes, and transporters but requires infrastructure, cost reductions, and training. The use of digital tools, AI-driven models, wearable biosensors, and real-time monitoring has potential in proactive care but needs to be validated, and newer treatments, including cannabinoids, anti-NGF mAbs, and Nav1.7 inhibitors, require long-term trials. To bridge the gap, policy change, education, and equity-based measures such as improved opioid regulation, reimbursement of necessary analgesics and adjuvants, national pain registries, low-cost regimens subsidized by the state, multidisciplinary care enabled through telehealth, and culturally modified assessment tools are necessary. Matching innovation with specific policy actions will make sure scientific progress leads to prompt, fair patient outcomes globally.

## Conclusions

The reviewed evidence highlights that CRP cannot be addressed with isolated or pharmacologic measures alone. It is multidimensional in that it is biological, psychological, and sociocultural, and thus demands the connection of mechanistic knowledge with the situation of a particular patient. Evidence-based interventions consist of the flexible application of the WHO analgesic ladder based on the mechanism of pain and patient objectives; early use of adjuvants (duloxetine, gabapentinoids, and corticosteroids) in neuropathic or mixed pain; interventional modalities (nerve blocks, neuraxial delivery, and RFA) in focal or refractory syndromes; palliative radiotherapy of bone and compressive lesions; and structured psychosocial interventions (CBT). Functional mapping and validated tools (e.g., FLACC and PAINAD) enhance the accuracy of assessment and customization of the treatment in pediatric and geriatric care. New modalities, such as pharmacogenomics, AI-based monitoring, and complementary therapies, such as selective use, have potential and need further validation.

Cost-effective interventions like immediate-release morphine with amitriptyline are comparable to their high-cost counterparts in LMICs. Policy reforms have made access to them easier, such as the 2014 opioid licensing reform in India, with no abuse. The multidisciplinary telehealth-supported care in sub-Saharan Africa and the national oncology pain curriculum in Uganda offer examples of scaling up low-cost treatment, policy, workforce, and digital health. Integration of mechanism-based strategies within resource-sensitive, culturally competent care pathways has the potential to revolutionize CRP management by shifting it to patient-centered, proactive care and enhancing pain control, treatment adherence, function, and quality of life across the globe.
